# Encountering staff-directed aggression within mental health and substance abuse services: exploring conceptions of practice following education

**DOI:** 10.1186/s13033-019-0277-8

**Published:** 2019-04-02

**Authors:** Erlend R. Maagerø-Bangstad, Knut Tore Sælør, Ottar Ness

**Affiliations:** 1 Faculty of Health and Social Sciences, Dept. of Health and Social and Welfare Studies, Centre for Mental Health and Substance Abuse, University of South-Eastern Norway, P.O. box 7053, 3007 Drammen, Norway; 2 Dept. of Education and Lifelong Learning, Norwegian University of Science and Technology, P.O. box 8900, 7491 Trondheim, Norway

**Keywords:** Staff-directed aggression, Prevention, Management, Practice, Mental health services, Substance abuse services, Education, Phenomenography

## Abstract

**Background:**

Staff-directed aggression represents a considerable concern in mental health and substance abuse services, and presents a substantial challenge to the quality and continuity of service provision for people with mental health and substance abuse problems. The practitioners themselves frequently request increased competence as a way to mediate the negative effects of staff-directed violence and aggression. The aim of this study is to explore how practitioners in municipal mental health and substance abuse services conceptualize practice in prevention and management of staff-directed violence. Furthermore, we seek to explore how these conceptions change following participation in two complementary and specially developed courses advancing respectively, qualified risk assessment and situational awareness and disempowerment-sensitive and de-escalation principles for practice.

**Method:**

The study was conducted by using a qualitative phenomenographic research approach. The data-material comprised two-step semi-structured interviews with ten participants from various parts of community mental health and substance abuse services in the Municipality of Oslo, Norway.

**Results:**

The analysis resulted in the development of five qualitatively different, hierarchically ordered, yet logically interrelated conceptual categories of practice concerning prevention and management of staff-directed aggression in community mental health and substance abuse services. These are: (1) *Observation, reporting and expectation of organizational intervention*, (2) *Application of personalized de*-*escalating skills and behaviour*, (3) *Delivery of team*-*based and standardized services*, (4) *Provision of perceptive and responsive services*, and (5) *Facilitation of sensitive, involving and reflexive care*. The categories vary according to the participants’ attentional focus on either the responsibility of the organization, staff members’ personally developed skills and techniques, team-based solutions, knowledgeable information processing in making professional judgements and reflexive, interpersonal service provision, as well as according to what meaning participants assign to practice. The authors have identified varying degrees of conceptual change following education in half of the participants.

**Conclusion:**

The results of this study both show that practitioners conceptualize practice in aggressive encounters quite differently. The study also indicate that it is important to consider participant awareness of the phenomenon of interest when devising educational activities for personnel in mental health and substance abuse services.

## Background

Generally, people with mental health or co-occurring substance abuse problems do not perpetrate violent aggression against others [1, 2]. Instead, people with mental health and substance abuse problems are more likely to become victims of violent acts than the population in general [3–5]. Yet, people with mental health or substance abuse problems are statistically more likely to engage in violent behaviour compared to the general population [2, 6, 7].

When persons with mental health or substance abuse issues do commit violent acts, the victims tend to be relatives of the perpetrator [2, 7]. Even so, staff in mental health services and services for people with co-occurring mental health and substance abuse problems, are frequently exposed to violent behaviour and aggression from service users, including physical attacks, violent threats and verbal abuse [5, 8, 9]. Violent incidents between staff and service users may have several adverse effects for service users, personnel and services alike, some of which include harm to the relationships between service users and staff [10], the quality of care [11], as well as the frequency and duration of home-visits [12]. In addition, incidents also frequently lead to involuntary hospitalizations of service users [7], and subsequent coercive measures [13].

Norwegian mental health and substance abuse services are largely interdisciplinary and comprised by both professionals and paraprofessionals [14]. Norwegian and international health-care policies regulating mental health and substance abuse services have, in recent years largely been redirected towards local, home-based, and patient-centred or person-centred care [15, 16]. However, research on violence in non-institutional, outpatient settings is only in its ‘infancy’ [17]. Hence, there are few prevalence-studies—or any other studies as far as we have been able to identify from the literature—on staff-directed aggression in Norwegian non-institutional mental health and substance abuse services. Albeit, in a recent national survey about 40% of learning disability nurses and social workers, one out of four care workers and nurses reported being a victim of either violence or threats at work during the preceding year [18]. In a report by the Norwegian Directorate of Health [19] 139 municipalities reported 111,376 incidents of threats and violence against staff or co-patients in municipal health services during 2012–2016.

A report on violence in Norwegian health care and social services [9] stated that competence development in prevention and management of client aggression was considered a desideratum by responding personnel for developing services apt to meet the challenges presented by aggressive encounters with service users. In 2010, the Agency of Health (AH) in the municipality of Oslo devised a project purposed at providing education for personnel in home-based mental health and substance abuse services. The project aimed at promoting competent, safe and persistent service provision for both service users and staff by making education in prevention and management of staff-directed violence available to the services. Additional supportive tools, such as pamphlets, guidelines and e-learning material was also provided by the project. These tools aimed at promoting and supporting continued competence and practice development among staff.

There are numerous ways of understanding violence and aggression [20], as well as accounting for the precursors for staff directed aggression in mental health care settings [8, 9, 13]. The view underlying the AH project was a disempowerment-sensitive perspective regarding aggression as stemming from persons’ attempts to master situations characterized by frustrating life conditions and structural impediments [21], situated within a disequilibrium of power between service providers and service users [22]. In this view, interpersonal factors, including staff demeanour and communication, have considerable influence on the prevention and management of aggression. Knowledge-based non-physical, de-escalation principles [23, 24], individual, as well as situational risk assessment [25] and recovery-oriented practices in mental health and substance abuse care services [26] underlies the education and tools made available for personnel through the project. The educational activities in the project have since 2010 been attended by over 3000 participants. In total 184 participants attended the two courses subject to the present study in 2017, named “*Basic course in management of threats and violence*” and “*Who am I in the encounter with the service user? Phase*-*oriented prevention and management of violence*”. The first course focused on emergency preparedness, situational and contextual awareness and risk assessment, while the latter course focused on disempowerment, interpersonal and relational dynamics and de-escalation principles. The organizers view the two courses as supplementary to each other, focusing on different aspects of practice in prevention and management of staff-directed aggression. These courses have played a foundational role throughout the AH project, as well as afterwards. Developing knowledge of how these courses have contributed to competence and practice development in the services has been a long-standing aim of the project, and the present study aims to identify some of the outcomes of the project.

The purpose of the current study is to explore and gain knowledge about how participants in the two complementary general courses conceive practice in encounters with staff-directed aggression, and how their conceptions develop following education. By this study, we aim to inform future education and training for staff in community based mental health and substance abuse services, by proposing insights from phenomenography in both designing and assessing the outcomes of education. In addition, we aim to contribute to the current knowledge base concerning non-institutional, staff-directed aggression. We developed two specific research questions for this study:

1. How do participants in the general courses in prevention and management of staff-directed violence and aggression conceptualize prevention and management practice in violent and aggressive encounters with service users?
2. How do participants’ conceptions of prevention and management practice change following education in basic and general principles of prevention and management of staff-directed aggression and violence?

## Methods

In this study, we have used phenomenography to explore descriptive categories of practice from interviews with participants in education. Additionally, by comparing participants’ responses prior to, and following education, we were able to assess changes in participants’ conceptions over time.

Phenomenography is a qualitative educational research approach aimed at describing the variation in individuals’ conceptions of the world, their awareness of and ways of experiencing a particular phenomenon [27, 28]. This research approach is a *second*-*order approach* [28], seeking to describe peoples’ experiences of phenomena in the world around them, based on a nondualist worldview: that there is only one (experienced) world and this world is constituted by an internal relationship between phenomena in the world and the persons experiencing them [28]. In phenomenography, “individual voices are not heard” ([28], p. 114) and hence, the description of variation is restricted to the collective level.

Phenomenography sets out to describe the experiential and conceptual aspects and boundaries of a phenomenon by applying the analytical features of ‘referential’ and ‘structural aspects’ to identify, respectively, the meaning of the phenomenon for individuals or how meaning is constructed and what part of the phenomenon is the focus of attention [28, 29]. Phenomenographic studies typically result in an ‘outcome space’, “the complex of categories of description comprising distinct groupings of aspects of the phenomenon and the relationships between them” ([28], p. 125). This outcome space is generally considered consisting of hierarchically inclusive, logically interrelated categories of description. This entails classifying conceptions, or ways of seeing, from less advanced to more advanced ways of seeing phenomena. This means that “higher-level” conceptions incorporate and add to the conceptions comparatively lower in the hierarchy. More advanced conceptions thus signify more complex and compound understandings of relevant or critical aspects of phenomena [27], and implies more competent and powerful ways to handle novel situations and encountered phenomena.

### Design

The design of this study is within a descriptive-explorative research framework. Phenomenographic research is from the outset an explicitly data-driven and empirically oriented research approach [30]. Yet, this study is informed by a social constructionist epistemology, viewing knowledge-production as communally based and scientific reasoning as inherently socially situated [31] and thus the authors acknowledge qualitative research as both an interpretative and co-constructionist enterprise. In the present study we have explored participants’ expressions of how they conceptualize their own practice, and attempted to present as representative an account of the participants’ expressions as possible. In this study, we sought maximization of experiential phenomenal variation and conceptualization among participants by employing, criterion-based, purposeful sampling [32].

### Recruitment and participants

We recruited ten participants registered to participate in one or both courses during the spring of 2017 in this study. All together 103 persons were registered for participation in the two courses. The participants in this study came from community mental health services, housing accommodation services for people with mental health or substance abuse problems and community and social welfare services. The age range was 23–53 years. The participants’ educational background and profession varied from no education to clinical mental health nursing. Relevant work experience ranged from 1 year to 20 years. We considered the relatively wide range of participant characteristics included in the sample to adequately reflect the heterogeneity of the staffing situation in Norwegian mental health and substance abuse care. All of the participants reported being victims of service user violence and/or violent threats at least once during their careers. Six of the participants also reported witnessing staff-directed violence or violent threats against colleagues. One of the participants revealed witnessing domestic violence during childhood and adolescence. Seven of the participants attended both courses. The other three participated in just one course, with two of them having participated in the other course at an earlier stage. This, we deemed to illustrate well the context and complexities of providing non-formal education to staff engaged in the day-to-day operations of their workplaces, limiting the opportunities of staff to attend several consecutive courses.

### Data collection

Qualitative semi-structured exploratory interviewing is the typical data-collection method in phenomenographic studies [29]. Data collection in this study was comprised of individual interviews prior to participation in courses and 4–6 months afterward. The interviews were conducted at the participants’ workplaces, except for one that was conducted at the first author’s workplace. The interviews were administered according to two corresponding interview guides, with the second interview guide additionally covering questions regarding the participants’ own perceptions of change. The interviewer initiated the follow-up interviews by giving a short oral summary of the participants’ answers from the first interview. The procedure both helped set the scene for participant reflection as well as provide an avenue for elucidating the researcher’s understanding of the first interview. The average length of the pre-education interviews was about 1 h. The follow-up interviews were on average an hour and a half in length. The interviews of ten participants, eight female and two male, were eventually included in this study, after we discarded three participants’ initial contributions due to either non-attendance in the education, difficulties in establishing contact in time for the follow-up interview or practical problems in administering the follow-up interview. All interviews were recorded and transcribed verbatim.

### Data analysis

There are different views on how to handle transcribed interviews within phenomenography. For this study, we favoured dealing with whole transcripts, in line with a contextualized view [33], over a decontextualized, fragmentary view of transcript analysis in the vein of Marton [27] and Marton and Booth [28]. This enabled us to maintain a contextual sensitivity during analysis, and allowed us to discern focal change in the participants’ conceptions over the course of the interviews, when comparing responses prior to and following education. Based on this, we applied Sjöström and Dahlgrens’ [34] description of a stepwise model of phenomenographic analysis in this study. These steps comprised: (1) the first author getting *familiarized* with the data by reading thoroughly through the transcripts, subsequently (2) *compiling* answers to particular questions, (3) performing *condensation*, or reduction of individual answers to more comprehensive statements, and (4) *grouping* similar statements in the material. All authors then collaborated in a (5) precursory *comparison* of categories, looking for similarities and discrepancies between the grouped meaning units, and in (6) *naming* categories, trying to find fitting expressions of their descriptive content. Eventually, all authors contributed in conducting a (7) *contrastive comparison* of the established categories, probing for potential content overlap. Upon venturing into exploration of instances of conceptual change in the data-material, we subsequently approached all 20 transcripts individually, by going into each transcript and identifying conceptions present therein. In line with the phenomenographic view of categories of description as hierarchically inclusive, logically interrelated, the expressions signalling the most advanced understanding in each interview eventually determined which transcript was assigned to which category. Finally, we summed up the categorizations of the prior- and post-education interviews separately to attain an impression of the conceptual change in the material.

Sjöström and Dahlgren [34] contend that research is difficult to describe in consecutive steps, referring to the mutual relationships between the various analysis steps. In agreement with this, Åkerlind [29] claims that phenomenographic analysis involves an iterative process of reading, re-reading, interpretation and revision. This was also how we experienced analysis in this study.

We pursued bracketing of researcher pre-conceptions by engaging in internal discussions and reflection between the authors of this study, as well as an iterative process of individual clarification of phenomenal awareness during analysis by the first author. The first is an example of dialogic reliability checking, which is one of the ways reliability is ensured in phenomenographic studies [29].

Running excerpts of transcripts and the preliminary findings through a reference group, and presenting and discussing the established categories of description with peers and other colleagues from the field, were additional measures for establishing pragmatic and communicative validity in this study. The reference group comprised two managers and two employees in municipal mental health services, two representatives with mental health or substance abuse service user experiences, and one representative from collaborating district psychiatric services in Oslo, in addition to the first author. We consulted with the reference group during the initial stages of the study, most notably to validate the interview guides, and in the end, to inform the finishing phases of data analysis. Parallel to Borg [35], the reference group members were first and foremost in an *advisory* position, never determining either research aims or the methodological approach, but nevertheless indispensable for ensuring researcher apperception of findings in relation to a local context and functioned as co-creators of knowledge in this study.

The final analysis resulted in five categories of description concerning the participants’ understanding of practice in preventing and managing aggression and staff-directed violence and/or violent threats.

### Ethics

The Norwegian Centre for Research Data granted recommendation for this study (Case No. 52044). Written and informed consent was obtained from each participant prior to the first interview. Each participant received written information upon first contact, and each pre-course interview were initiated with the interviewer repeating the same information orally. Special emphasis was put on the ethical principle of voluntary participation and the possibility of withdrawing from the study without any repercussions for the participants. The question of confidentiality was particularly important for us since all but one interview were conducted at the participants’ workplaces. We have ensured confidentiality in this study by anonymizing all information about the participants and made sure not to include any information that might have contributed to disclosing the identity of the participants.

## Results

In the following section, we present the identified descriptive categories, followed by a brief summary of the detected patterns of change over the course of the interviews, as they were ascertained following comparisons of interview-responses prior to and after participation in the courses. We represent each conceptual category by providing illustrative quotations from the interviews in the following section.

### Descriptive categories

Analysis resulted in a hierarchical outcome space consisting of five qualitatively different conceptions of handling and managerial practice in situations with staff-directed aggression. The first four represent conceptions of practice centred on aspects of staff skills and strategies, while the latter focuses on the service user-staff relationship. In Table 1, we present the outcome space from this study.

**Table 1:** Outcome space of participant conceptions of practice in staff prevention and management of staff-directed aggression and violence

Descriptive categories	Referential aspect	Structural aspect
1. Observation, reporting and expectation of organizational intervention	Safe-guarding the boundaries of services and maintaining staff safety	Focus on organizational responsibility
2. Application of personalized de-escalating skills and behaviour	Finding practical and applicable solutions without access to a fixed set of methods and strategies	Focus on staff’s idiosyncratic abilities, prerequisites and techniques
3. Delivery of team-based and standardized services	Seeing the need for systematic problem-solving in the handling and management of risk	Focus on cooperation and support between experienced and skilled colleagues
4. Provision of perceptive and responsive services	Recognizing service users as sources of valuable information in making sound professional judgements	Focus on communication with service users and abilities for employing informed service-provision by staff
5. Facilitation of sensitive, involving and reflexive care	Professional practice as attentive of human complexity and valuing interpersonal relations	Focus on service users and staff as persons, and reciprocal connectivity

#### Observation, reporting and expectation of organizational intervention

In the first category, participants conceive practice in encounters with staff-directed aggression in terms of “Observation, reporting and expectation of organizational intervention”. Participants expressing this conception focused on the provision of information on service user behaviour and symptomatology as the basis for expected external intervention and management. Conceptions of staff responsibilities were restricted to primarily ensuring a safe continuation of services “as-they-were”, and possibilities for constructive interaction with service users were limited. In this conception, valued personnel skills in service provision were experience-based alertness and risk-assessment, as well as perspicuous, predictable and safeguarding demeanour in practice toward service users. Insecure staff demeanour carried potential for pre-dispositioning of threatening or violent interactions between staff and service users:

*“If I wind up in a setting with a service user where I am, in some way going to impart something and give the impression that I am very uncomfortable and insecure, then I think this will trigger many who may be manipulative or violently predisposed to be intimidating or, in a way, to try to acquire something…”.* (M1, first interview)

Participants viewed management of service user violence to be primarily the responsibility of superior parts of the community mental health service-system. Handling and management of staff-directed aggression and violence consisted of authoritarian, hierarchical sanctions such as eviction, time-limited discontinuation of services or replacement in other mental health or substance use services: “*The concept here is basically zero*-*tolerance for violence. If you are violent, then you’re in the wrong place”.* (M1, first interview)

Participants frequently mentioned the negative impact of economic priorities and perceived systemic renunciation of responsibilities, particularly when they perceived their expectations of organizational intervention as unfulfilled.

In this conception, the workplace was experienced as being ripe with risk and consisting of high-risk users. Professional detachment and personal fortitude were means of protecting the staff’s physical and mental health in these conceived volatile and unpredictable circumstances, and service user perspectives were in this view perceived as being largely insignificant for the prevention and management of staff-directed violence in services.

#### Application of personalized de-escalating skills and behaviour

In the second category describing conceptions of practice, “Application of personalized de-escalating skills and behaviour”, participants emphasized idiosyncratic and personally developed modes of handling and management of threatening or violent interactions between staff and service users. In this view, preventive and management practice were largely ad-hoc and often manifested a more common sense ‘guesstimate’ of what works, or a more or less compliant adaptation of established workplace procedures. Even so, participants asserted recognition of de-escalation as the primary and most beneficial strategy in prevention and management of staff-directed aggression, albeit argued for in a largely unfounded manner. In the perceived absence of a fixed set of techniques and strategies, participants viewed the staff’s personal risk-negotiating skills and aptness in heated exchanges with service users as key factors in resolving and handling risk at work:

*“… I try to meet the user where he, in a way is* […]*. I try to see “eye to eye”* [with the service user] *… I am also very solution oriented and I do not see things in a single*-*minded manner and I do not always have to go by the book. I know that it can be very provoking for some service users. When staff do not go “out of the box” at all, it can be very provoking. I have round edges, so I do not think that I am a source of conflict”.* (M2, first interview)

Participants who initially expressed this viewpoint described their competence as fleeting, wanting or irrelevant. Reflections concerning their own practice were frequently indecisive and ambiguous, and compliance with practices in their workplaces were found to be one way in which staff resolved this uncertainty, although some expressed concern or ambivalence regarding workplace practices and routines:

*“Occasionally, I have, in some way, wondered if we are somewhat cowardly in here. Are we avoiding… Are we being perceived as yellow*-*bellied because we are so readily withdrawing when things may get a little difficult?” (F1, first interview)*

Ambivalence and uncertainty according to workplace practices were largely left unresolved due to a lack of understanding of more appropriate measures of violence prevention and management practices.

#### Delivery of team-based and standardized services

In the third category, “Delivery of team-based and standardized services”, the practitioners emphasized the significance of the team and the establishing of informed routines and practices at the workplace as essential in preventing and managing staff-directed aggression. Expressions of this form of understanding carried the implication of knowledgeable and experience-based practice, embodied through either repetition or some sort of training. In this view, the team is the primary locus for proficient handling of staff-directed aggression and violence. Participants expressing this view considered aggression largely as an occupational problem to be solved within a system of established and consistent practices and routines, supporting individual and team-based collaborative interventions. The standardization of services signified principles of intervention and management of aggression agreed upon by the team. Participants held the team high in regard as both a source of support as well as origination and implementation of congruent and authoritative interventions in situations of aggressive or threatening behaviour from service users:

*“I think we are doing many things very well already because we report back when we observe changes* (…)*. What are we going to do?” And then we talk and reach an agreement about going in pairs, talk about it* [with the service user]*, ask him to come in for a consultation, bring the manager in, avoid home*-*visits, for example. For a while. Or if we are just going to have contact with him by phone. We come to a conclusion, and then we choose a strategy”.* (F2, first interview)

Participants viewed reciprocal relationships, openness, accountability and trustworthiness between colleagues as prerequisites for cooperation:

*“… if we are going to have a good work environment and find satisfaction in the work we are doing, from our own prerequisites and from within the boundaries we work, I think it is kind of a continuous process among us colleagues, that we are, in a way trying to do the best we can. By this, I mean talking to each other and maybe being open with one another, trusting each other and everything”.* (M1, second interview)

This conception placed the individual staff member as responsible for contributing to the workplace discussions, and should accordingly be able to expect colleagues and managers to pay attention to their experiences and opinions, when the team identified strategies for prevention and management of staff-directed violence. Some spoke of an interplay between knowledgeable and trusted colleagues, approximating a theatrical performance during aggressive interchanges, while others put forth a more systematic and reflective dialogue with other team members as key in developing adept practice in their workplaces.

#### Provision of perceptive and responsive services

In expressing the fourth conception of practice, “Provision of perceptive and responsive services”, participants embraced information and observations gained from service users to be imperative in establishing sensible and well-founded services sensitive to the prevention and management of staff-directed violence. Staff needs to be perceptive and alert in making judgements concerning their work, but is still the authoritative factor in employing preventing and managing strategies, according to this conception. The view of professional practice was that it is based upon experienced judgement and monitoring of situations, the availability of tools and strategies, and the ability to adjust one’s own demeanour and practices according to the available information. Participants perceived the professional-personal boundary as a phenomenon to be negotiated in dialogue with the service users. Knowledge of the individual service user and her idiosyncratic triggers and vulnerabilities were considered to be pivotal in making judgements for expedient practice by staff. On the other hand, participants viewed service user-staff relations as presenting a challenge in divulging staff vulnerabilities:

*“But if there is a client that I have dealt with extensively, you know, have had many discussions with, a great deal of “hot battles”, so to speak… then he has been accustomed to how I react and what I look like when I am scared or angry…”.* (F6, first interview)

Participants viewed routines and regulations as supportive tools in the process of inferring viable courses of action. Sound implementation of workplace routines emerge on the basis of staff knowledge about violence and potentially perilous situations.

Dialogue with service users following incidents came out as an important avenue for learning and subsequent practice development:

*“… I think that in the aftermath of a situation that has escalated and possibly become an aggressive interaction or something like that… When things have settled, one can go through the situation together with the service user with care to what has… how we experienced it and how he experienced it, or whatever lay behind it and how we can try to prevent it from happening again”.* (F5, second interview)

Although deemed important, participants perceived the follow-up of the service user after aggressive episodes in a somewhat technical and detached manner. Participants expressing this conception, portrayed staff as being primarily responsible for acquiring valid information to be used in judging appropriate preventive and managing responses towards the service users.

#### Facilitation of sensitive, involving and reflexive care

In this study, this last conception represented the most complex and compound understanding of practice. By viewing practice in terms of “Facilitation of sensitive, involving and reflexive care”, participants expressed valuation of equality between service user and personnel in the service relationship. In addition, they afforded particular significance to service users’ opinions, resources and experiences when outlining low-conflict service-provision. This appears to be a considerate and person-centred stance to collaboration, sensitive to the interconnectedness and personhood of both the service users and staff. This conception entailed respect and positive attitudes and sometimes, even affection as pivotal for preventive practices with service users:

*“I actually have a lot of respect and I am very fond of them, and that is… They are basically good people and maybe they recognize that in me”.* (F3, first interview)

Instead of condemning aggressive and threatening behaviour from service users, participants conceived it as communication of disempowerment and frustrating circumstances in life. Participants perceived that such communication ought to be met with heightened levels of care and attentiveness towards service users, instead of punitive responses.

Personnel responsibilities include accommodating for empowering, amenable and user-friendly services and professional helping relations. Boundaries between the private and the personal appeared somewhat indistinct when participants talked of expressing personal experiences as a basis for communicating empathetic understanding of the other’s position. Staff behaviour and demeanour, were viewed by participants as means to model desired and appropriate intrapersonal communication between personnel and service users.

Practice, in this conception was continuously subject to reflection, both individually and between colleagues. Hence, the depiction of practice was that it is an exacting undertaking demanding ample mental resources, attentiveness and flexibility from personnel:

*“*[…] *even though I am myself with each of these* [the service users]*, I am different as well because they all have different boundaries too, and you can go from one apartment and joke and fool around, and everything is swell, and then you go to another and then you are more quiet and calm. So things change quickly in this line of work”.* (F7, second interview)

Instances where mental resources of staff are temporarily confined or unstable, caused by, for instance, stressors from the staff members’ own private lives or periods of weariness, required pre-emptive measures, such as time-limited withdrawal from the most demanding interactions or mindful abandonment of one’s own problems at home while at work:

*“If we carry something that bothers us, we have to leave it behind at home because they* [the service users] *have enough on their plate. That is why they are here where we work in the first place. So I think… and that is when we have to be professional and that is strenuous”*. (F3, first interview)

This conception, in a sense, summed up the preceding conceptions by encompassing systemic, contextual, individual and interpersonal aspects of practice, and subsequently transcended them by sensitively acknowledging both parties’ contributions in solving an aggressive interchange.

### Expressions of change following participation in courses

Half of the participants expressed some form of change in their conception of practice in volatile situations with service users, whereas the other half expressed no such change. Although a majority of the participants in this study expressed some perceived impact from the courses, only half of them voiced conceptions that the researchers eventually deemed salient enough to label them expressions of actual, qualitatively advanced conceptions. We have depicted the identified conceptions prior to and following the courses in Table 2.

**Table 2:** Expressed conceptions identified prior to and following participation in courses

Conception	Pre-courses	Post-courses
1	I	–
2	III	–
3	III	IIII
4	I	III
5	II	III

Identified patterns of change following courses: from conception (conc.) 1 → conc. 3 (two steps): one instance, from conc. 2 → conc. 3 (one step): one instance, from conc. 2 → conc. 4 (two steps): one instance, from conc. 2 → conc. 5 (three steps): one instance, from conc. 3 → conc. 4 (one step): one instance

Whereas four participants articulated the two initial and most rudimentary conceptions in their first interview, none maintained these during the second interview.

None of the participants conceptualized practice in a way that could have been associated with moving from a higher to a lower level of understanding, although this movement would also have been conceivable.

Patterns of change in the data material involved movement from one category to the next or from one category to the second succeeding category, and we even identified one incident of three-step change (moving from the second conception to the fifth) in the material.

## Discussion

This study aimed at exploring how staff in mental health and substance abuse services conceptualized their own practice in prevention and management of staff-directed aggression and how these conceptions changed following participation in education in prevention and management of staff-directed aggression. The five qualitatively different conceptions of practice we developed from our data differed significantly according to both their levels of complexity and their structural and referential aspects. The logical inclusivity of the hierarchical outcome-space was manifest in this study when participants, voicing higher-order conceptions, concomitantly referred to aspects in line with other, comparatively subjacent conceptions over the course of an interview.

As we have described earlier, there is a substantial gap in the literature concerning how practitioners in non-institutional, home based mental health and substance abuse care settings conceptualize practice in encounters with staff-directed aggression. As we have mentioned, Campell [17] have labelled the whole field of research on staff-directed, non-institutional violence and aggression as being in its’ ‘infancy’. Given our aim to contribute to the current knowledgebase on the topic, we will therefore commit ourselves in the remainder to primarily discuss the implications of our findings, as well as provide an argumentation on how education might have contributed to bring about the observed conceptual change in participants.

Sandberg and Targama [36] have outlined an interpretative approach to competence, emphasizing understanding as essential for practice and competence-development. Inextricably linked to a person’s understanding of work is the particular knowledge, skills and attributes apparent in her understanding thus determining her performance. Underlying this view is the notion that some understandings are viewed as normatively better, i.e., more functional than others, parallel to the notion of ‘more powerful ways of seeing’ put forth by Marton and Booth [28] and Marton [27].

Participants voicing conceptions in line with the first, most rudimentary category, expressed expectance of intervention from external and hence more unaccountable agencies meanwhile focusing on safeguarding, protective measures in their practice. Aberhalden et al. [13] links a paternalistic model of care with inattentive, coercive and controlling practices toward service users. Björkdahl, Palmstierna and Hansebo [37] have depicted similar safeguarding practices as “bulldozing” and have argued this to be a common way personnel in mental health settings protect themselves against inpatient aggression. The participants voicing such sanctioning strategies in our material seems to condone such practices as one way to ensure a safe working environment. Albeit, service users may experience such practices as staff-initiated, aversive stimulation, thus heightening the potential for reactive aggression toward staff [38, 39]. Adding to the negative impact of safeguarding practices, Whittington [40] have also linked burnout among mental health personnel to low tolerance for service user aggression and associated negative attitudes.

The focal awareness in the consecutive categories integrate accumulating aspects of staff knowledge, skills and attributes, placing the practitioners increasingly at the centre of practice. Simultaneously, we see increasing levels of awareness of the other parties involved in practice. This increase implies among other things, a movement from an indistinct view of the working community to colleagues and the team having gradually increasing importance for the execution of their practice, the understanding of practice and the development of practice. Sandberg and Targama [36] argues that socialization into a shared understanding is a collective avenue of practice and competence development in the workplace. This implicates a more conducive approach for designing education for personnel by taking the workplace culture and the collective level of understanding in a workplace into account at early stages of the planning. From the third category, it is evident that the team carries additional significance by providing support for team members. Research indicates that social support between colleagues are protective factors against both workplace stress and negative job performance [41], as well as being predictive of workplace violence victimization [42] and promoting well-being in staff following violent assaults [43].

A majority of the participants in the study judged experience as crucial for understanding staff-directed violence and for knowing themselves and their typical reactions in aggressive encounters with service users. Sandberg and Targama [36] have proposed a view of conceptual change that considers the individual’s experience and her efforts to make meaning reflectively of these experiences, as essential for conceptual change. This experiential factor might also apply to personal experiences with violence outside of work, which at least one of the participants had experienced. Sundberg [44] models professional competence and practice as partly contingent on both the individual’s personal and professional experiences, while Dall’Alba and Sandberg [45] places emphasis on ‘lived experience’ as a basis for professionals’ competence. Experience with aggression, individual phenomenal awareness and collegial reflection thus seems to be a necessary pre-requisite for competent practice. The educational implications for this might be to devise follow-up sessions after participation in courses or advising case-based learning by discussions and reflections in the workplace following education to ensure the establishment of connections between participants’ workplace experiences and the learning material.

In the two latter categories, participants gradually focus their attention on the service user, either as a source of information or as an equal partner in the outlining of services. The ‘proficient’ practitioner in Benner’s [46] ‘novice-to-expert’ model makes use of experience-based anticipative knowledge, perceives situations as wholes and allows her performance be guided by maxims. Judgements for application of maxims require an in-depth understanding of the particular situation. This involves what Tanner, Benner, Chesla and Gordon [47] termed ‘knowing the patient’, and in particular, knowing her particular patterns of responses and the nature of her problems, thus enabling the provision of responsive services, minimizing conflict, and potentially maintaining both service user and personnel well-being and serving both parties interests in the situation. Participants seemed to subscribe to this notion when they expressed knowledge of the individual service-user and reflective practice as essential in outlining safe and persistent service provision.

By valuing and accommodating for involvement and shared decision-making in outlining practice and reciprocal relationships characterized by acceptance, respect, engagement and compassion, participants in the latter category describe their practice in alignment with principles described in a person-centred practice view [48], as well as recovery-oriented approaches in mental health and substance use care [49]. Biringer et al. [50] have shown recovery-based practice to be both supportive of advancing persons with mental health problems’ understanding of themselves and their own problems, as well as to change their feelings and behaviours. Roth and Crane-Ross [51] found that service user perceptions of having influence on service-related decisions have an effect on mental health outcomes. According to Borg and Kristiansen [49], helpful relationships are in part, marked by willingness by the professional to view the other as resourceful and capable and in addition, willingness to engage in reciprocal self-disclosure. Person-centred practice, on the other hand, contributes to service users experiencing respect, interest, attention, confidence and acceptance of shared responsibilities and control in healthcare relationships [52]. In light of a disempowerment-sensitive perspective on aggression, this implies that collaborative, recovery-based and person-centred practices are conducive of atmospheres where aggression and violence towards staff is likely to be deemed unwarranted by service users.

### Conceptual change

Most of the participants in the study expressed some level of perceived impact from participation in the courses, mostly in terms of the course content reminding them of some areas of practice they had grown less cognizant of or as contributing to (re)establish awareness of the phenomenon as a pressing issue to manage at work. Differences in levels of awareness between the participants seems to be a plausible explanation of why some participants experienced significant conceptual change whereas others experienced none.

Healthcare workers frequently perceive staff-directed aggression as being “part of the job” [53]. All of the participants in this study shared experiences of victimization from work-place violence and a majority reported witnessing this happening to their colleagues. Although a majority described occurrences of staff-directed aggression appearing on an intermittent basis, some described themselves as working in high-risk environments with recurrent encounters with service user aggression. Needham [54] suggests habituation as a perceptive mechanism in psychiatric nurses’ experiences with patient aggression. This might also explain why some were not sufficiently aware of workplace violence and the need to develop practice in prevention and management of staff-directed aggression.

Marton and Booth [28] and Marton [27] point to Gurwitsch’s concept of ‘thematic field’, in explicating the variation in awareness between people. The thematic field depicts how some aspects of a particular phenomenon are focused while other are held in the focal background, while other aspects might be held in focus at other times and in other situations. This signals that a phenomenon might undergo a range of qualitative experiential variation for a person over time. Conceptual change implies focal change and subsequently, the ability to discern relevant aspects of the phenomenon. When situations change, the structure of our awareness is also likely to change and thus, people are able to focus on different aspects of the phenomenon. Such change in the thematic field are brought about by, for instance, participation in education, individual or collegial reflection on course-material, experiences with the phenomenon on the job, or even, participation in interviews.

Schön [55] have described how the reflective practitioner engages in tacit, embodied and conscious selective processing of data and experimentation with data at work as a basis for judging appropriate responses to different situations. The participants expressing conceptions in line with the fifth category, conveyed reflection-in-practice as an arduous, yet necessary condition for qualified practice in challenging situations. Other participants spoke of “sensing” or having a tacit, embodied awareness of risk and assessment of situations. Lillevik and Øien [56] has identified “milieu sensitivity” as one important factor in violence-preventive practice in childcare institutions, described as an awareness towards interactional qualities and sensitivity of ward atmosphere. Similarly, Ervik et al. [57] have found application of “antennas” to be one way in which personnel in low-threshold housing facilities for people with substance use problems describe their non-verbal, interactional competence in cooperating with service users. In light of this, it seems reasonable to suggest that both conscious conceptualization as well as experiential and embodied awareness are significant for practice. The educational implications of this might be to include training and simulations capable of eliciting both conscious deliberation as well as emotional and physical awareness with participants in education.

### Limitations

It might be plausible that a more sizable sample could have spawned other categories of description than those identified in this sample. A further limitation is that we, by the chosen sampling procedure have not considered conceivable variations in the participants’ workplace environments and cultures, potentially omitting an important influence on participant conceptions. The results, we propose, are still both locally significant and relevant as well as meaningful to the phenomenon under scrutiny and might withal contribute to the understanding of both competence, practice and similar educational situations and contexts. Finally, whilst we have argued for the advantageousness of the most advanced conceptions, none of the implied practices have been empirically tested with regard to efficiency in actual prevention and management of staff-directed aggression. However, we find the correspondence between the three highest ranked conceptual categories in this study and contemporary recommendations and guidelines in prevention and management of aggression noteworthy.

## Concluding remarks

We suggest that the finding in this study best be viewed as a contribution to the continuous exploration of the different ways the phenomenon of practice in staff-directed aggression is experienced, thus adding to the “collective mind” concerning the phenomenon [58] as well as indicating a contribution from the offered education on participants’ conceptions of own practice.

The patterns of conceptual change in participants identified in the study, we find to be supportive of a discernible contribution from the courses in advancing conceptions in line with the third, fourth and fifth category, being that these conceptual categories are closely associated with the knowledge-based, collaborative, disempowerment-sensitive and recovery-oriented learning-material offered through the courses. Furthermore, we have found that the participants initially voicing the two less advanced and conceivably, most disadvantageous conceptions expanded their understanding of practice by the second interview. This finding arguably indicates a beneficial development for both the practitioners, by avoiding potentially unsafe and unwarranted practices, as well as for the service users whom they serve, by potentially enhancing the quality of care and lowering the level of conflict during the provision of services. This highlights how systematic education might enable reflective practices concerning service user aggression for personnel in mental health and substance use services.

This study describe both practitioners’ varying understandings and thus, implementations of practice, as well as carry implications on how to develop education and training in mental health and substance abuse services. It is critical in an educational setting to explore how participants assign meaning and focal structure to their experiences of the topical learning objective, to adjust teaching efforts and accommodating courses supportive of the educational intention. There is evidently substantial variation in understanding and practice, with the more comprehensive and ‘powerful ways of seeing’ being inextricably interrelated with less comprehensive conceptions. To support practice development and sustainable services for service users, educational personnel, agencies and managers of services have to be mindful of this variation and consider how it might affect participant learning when administering education and training for personnel. This would implicate the need for a more thorough mapping of participant conceptions prior to participation in education, followed by a necessary outlining of the subsequent education structured according to the present phenomenal conceptions and experiences of the learning objective among the participants. In actual teaching situations, phenomenography advises the application of the present conceptual variation among learners as a mechanism through which awareness of various aspects of the phenomenon are brought to their attention, thus potentiating the discernment of more critical aspects of a phenomenon [27, 28]. This would require engaging the participants to share views and experiences with each other during education.

As we have identified, staff-directed non-institutional aggression is an understudied area, and based on our findings we suggest the possibility of empirical testing of the established categories and implied practices might provide an avenue for additional inquiries into the phenomenon of violence prevention and management practice. One way to do this could be to test the various practices against various levels of staff-directed aggression in services to establish an empirical basis for recommended practice. Finally, a requirement for a more elaborate description of the phenomenon of practice in prevention and management of staff-directed aggression would also be to encompass the perspectives and experiences of service users.
